# Supplementary material for: Mechanistic Modelling and Bayesian Inference Elucidates the Variable Dynamics of Double-Strand Break Repair
Source: PLoS Comput Biol. 2016 Oct 14;12(10):e1005131. doi: 10.1371/journal.pcbi.1005131 (PMC5065155; doi:10.1371/journal.pcbi.1005131)
Supplement: S1 Text — contains: a full description of the dynamical model; a full description of the Bayesian hierarchical model; details of the density-weighted integral approach and expressions for our approximate DIC and BIC model comparison analysis. (PDF) [file pcbi.1005131.s001.pdf]

Mechanistic modelling and Bayesian inference elucidates the variable dynamics of double-strand break repair.

M. Woods and C.P. Barnes

SUPPLEMENTARY INFORMATION

1 Overview of the modelling approach

We have eight datasets $D = \{D1, D2, ..., D8\}$ which represent the repair kinetics in wildtype cells, genetic knockouts, and particular stage of the cell cycle. We wish to fit a model to these data simultaneously to infer the number of underlying repair processes plus their dynamical properties. To achieve this, we have further developed the software ABC-SysBio [1,2] to include an option to perform ABC SMC on a hierarchical model structure. In our hierarchical framework, we wish to obtain K_i^d for a number of processes, where i represents the repair process and $d \in \{1, ..., 8\}$ denotes the dataset. These parameters are in some sense nuisance variables, with "true parameters" (or parameters of interest) of the repair process μ_i, which represent the means of lognormal distributions and σ the variance (we group these hyper parameters as the vector $\gamma = (\mu_i, \sigma)$). The K_i^d are drawn from these population level distributions. In this case, the joint density can be written

$$p(D, K, \gamma) = p(D|K)p(K|\gamma)p(\gamma) \tag{1}$$

where Bayes rule becomes

$$p(\gamma|D) = \frac{p(\gamma)\int p(D|K)p(K|\gamma)dK}{p(D)}. \tag{2}$$

This is the posterior of the hyper parameters given the data, D. The integral indicates that we sum over (marginalise) the K values. We can include this into ABC by simulating data D^* using the following scheme:

$$\mu \sim U(\alpha, \beta)$$
$$K \sim LN(\mu, \sigma)$$
$$D^* \sim f(D|K)$$

In our study, $f(D|K)$ is the data generating model, and is a realisation of the stochastic reaction system described below.

2 Biochemical kinetic models

2.1 One, two and three process models

To investigate the minimum number of repair processes that can explain the data, we consider models of the form

$$x + E_i^d \xrightarrow{K_i^d} y_i \tag{3}$$

$$y_i \xrightarrow{K_i'^d} \emptyset + E_i \tag{4}$$

where x represents a double-strand break (DSB), K_i^d and E_i^d are the parameter and recruitment protein for dataset d and repair mechanism i. Note that we have used the empty set notation, $\emptyset$, to make explicit that the DSB has been repaired and the recruitment protein released. Each recruitment protein has a conservation equation

$$E_i^d + y_i^d = C_i^d, \tag{5}$$

where C_i^d is the total amount of recruitment protein for dataset d and repair mechanism i. If for any mechanism, the first protein to bind is repressed, then we remove all reactions corresponding to that mechanism. If a protein downstream of the first protein to bind is repressed, then we remove the reaction corresponding to end ligation for that mechanism. We compared three different models $M_1 : \{i = 1\}$, $M_2 : \{i = 1, 2\}$ and $M_3 : \{i = 1, 2, 3\}$. For the model M_2, we assume there are only two processes of non homologous end joining (NHEJ) and an alternative, such that we group single strand annealing (SSA) and alternative end joining (A-EJ) together as one alternative process.

2.2 Three process knockout model

Our final model consists of two reactions for each repair mechanism, giving a maximum total of six reactions for each data set:

$$x + E_1 \xrightarrow{K_1} y_1 \tag{6}$$

$$y_1 \xrightarrow{K_1'} \emptyset + E_1 \tag{7}$$

$$x + E_2 \xrightarrow{K_2} y_2 \tag{8}$$

$$y_2 \xrightarrow{K_2'} \emptyset + E_2 \tag{9}$$

$$x + E_3 \xrightarrow{K_3} y_3 \tag{10}$$

$$y_3 \xrightarrow{K_3'} \emptyset + E_3 \tag{11}$$

Each dataset, d, has three conservation equations

$$E_1^d + y_1^d = C_1^d \tag{12}$$

$$E_2^d + y_2^d = C_2^d \tag{13}$$

$$E_3^d + y_3^d = C_3^d. \tag{14}$$

When the reactions are taken to be deterministic, the wild type system can be described by the following set of nonlinear ordinary differential equations.

$$\frac{dx}{dt} = V - x(K_1 E_1 + K_2 E_2 + K_3 E_3) \tag{15}$$

$$\frac{dy_1}{dt} = K_1 E_1 x - K_1^{'} y_1 \tag{16}$$

$$\frac{dy_2}{dt} = K_2 E_2 x - K_2^{'} y_2 \tag{17}$$

$$\frac{dy_3}{dt} = K_3 E_3 x - K_3^{'} y_3 \tag{18}$$

where V is the initial concentration of DSBs. This system of equations can be solved numerically, however due to the low numbers of DSBs, probabilistic effects need to be correctly accounted for, so we use a stochastic formalism. To determine the most probable set of parameters to give rise to the experimental data - termed the posterior distribution - we apply approximate Bayesian computation sequential Monte Carlo (ABC SMC) [3,4].

3 Initial conditions and priors

The prior distributions for the hyperparameters were fixed across all datasets. In principle the recruitment protein numbers, C_i, can be inferred from the data. However we found little difference in the posterior distributions when fixed, so we chose to reduce the number of free parameters.

3.1 One process

Hierarchical priors: $\mu_1 \sim U(-4,4)$, $\mu_2 \sim U(-4,4)$, $\sigma \sim U(0.05, 0.9)$.

constant: $C_1 = 700$, $C_2 = 700$, $C_3 = 700$, $C_4 = 2800$, $C_5 = 2800$, $C_6 = 1906$, $C_7 = 1814$ and $C_8 = 1128.7$.

Recruitment protein initial conditions: $E_1^1(0) = 700$, $E_1^2(0) = 700$, $E_2^2(0) = 700$, $E_1^3(0) = 700$, $E_2^3(0) = 700$, $E_1^4(0) = 2800$, $E_2^4(0) = 2800$, $E_1^5(0) = 2800$, $E_1^6(0) = 1906$, $E_1^7(0) = 1814$, $E_1^8(0) = 1128.7$.

State vector initial conditions: $x^1(0) = 700$, $y_1^1(0) = 0$, $x^2(0) = 700$, $y_1^2(0) = 0$, $y_2^2(0) = 0$, $x^3(0) = 700$, $y_1^3(0) = 0$, $y_2^3(0) = 0$, $x^4(0) = 2800$, $y_1^4(0) = 0$, $y_2^4(0) = 0$, $x^5(0) = 2800$, $y_1^5(0) = 0$, $x^6(0) = 1906$, $y_1^6(0) = 0$, $x^7(0) = 1814$,

$y_1^7(0)=0$, $x^8(0)=1128.7$, $y_1^8(0)=0$.

3.2 Two process

Hierarchical priors: $\mu_1 \sim U(-4,4)$, $\mu_2 \sim U(-4,4)$, $\mu_3 \sim U(-4,4)$, $\sigma \sim U(0.05,0.9)$.

constant: $C_1=700$, $C_2=700$, $C_3=700$, $C_4=2800$, $C_5=2800$, $C_6=1906$, $C_7=1814$ and $C_8=1128.7$.

Recruitment protein initial conditions: $E_1^1(0)=700$, $E_2^1(0)=700$, $E_1^2(0)=700$, $E_2^2(0)=700$, $E_1^3(0)=700$, $E_2^3(0)=700$, $E_1^4(0)=2800$, $E_2^4(0)=2800$, $E_2^5(0)=2800$, $E_2^6(0)=1906$, $E_2^7(0)=1814$, $E_1^8(0)=1128.7$, $E_2^8(0)=1128.7$.

State vector initial conditions: $x^1(0)=700$, $y_1^1(0)=0$, $y_2^1(0)=0$, $x^2(0)=700$, $y_1^2(0)=0$, $y_2^2(0)=0$, $x^3(0)=700$, $y_1^3(0)=0$, $y_2^3(0)=0$, $x^4(0)=2800$, $y_1^4(0)=0$, $y_2^4(0)=0$, $x^5(0)=2800$, $y_1^5(0)=0$, $x^6(0)=1906$, $y_1^6(0)=0$, $x^7(0)=1814$, $y_1^7(0)=0$, $x^8(0)=1128.7$, $y_1^8(0)=0$, $y_2^8(0)=0$.

3.3 Three process

Hierarchical priors: $\mu_1 \sim U(-4,4)$, $\mu_2 \sim U(-4,4)$, $\mu_3 \sim U(-4,4)$, $\mu_4 \sim U(-4,4)$, $\sigma \sim U(0.05,0.9)$.

The total amount of protein and initial conditions were set according to the data.

constant: $C_1=700$, $C_2=700$, $C_3=700$, $C_4=2800$, $C_5=2800$, $C_6=1906$, $C_7=1814$ and $C_8=1128.7$.

Recruitment protein initial conditions: $E_1^1(0)=700$, $E_2^1(0)=700$, $E_3^1(0)=700$, $E_1^2(0)=700$, $E_2^2(0)=700$, $E_3^2(0)=700$, $E_1^3(0)=700$, $E_2^3(0)=700$, $E_3^3(0)=700$, $E_1^4(0)=2800$, $E_2^4(0)=2800$, $E_3^4(0)=2800$, $E_2^5(0)=2800$, $E_3^5(0)=2800$, $E_2^6(0)=1906$, $E_3^6(0)=1906$, $E_2^7(0)=1814$, $E_1^8(0)=1128.7$, $E_2^8(0)=1128.7$.

State vector initial conditions: $x^1(0)=700$, $y_1^1(0)=0$, $y_2^1(0)=0$, $y_3^1(0)=0$, $x^2(0)=700$, $y_1^2(0)=0$, $y_2^2(0)=0$, $y_3^2(0)=0$, $x^3(0)=700$, $y_1^3(0)=0$, $y_2^3(0)=0$, $y_3^3(0)=0$, $x^4(0)=2800$, $y_1^4(0)=0$, $y_2^4(0)=0$, $y_3^4(0)=0$, $x^5(0)=2800$, $y_2^5(0)=0$, $y_3^5(0)=0$, $x^6(0)=1906$, $y_2^6(0)=0$, $y_3^6(0)=0$, $x^7(0)=1814$, $y_2^7(0)=0$, $x^8(0)=1128.7$, $y_1^8(0)=0$, $y_2^8(0)=0$.

4 Details of the ABC inference

Reaction systems were generated in the software COPASI [5] and used as input files in a hierarchical implementation of the software ABC-SysBio [1, 2] (all code and model files are available in the ucl-cssb GitHub

repository). ABC-SysBio implements the method of sequential importance sampling. This approach proceeds by the repeated sampling of parameters (particles), simulation of the model, and comparison of model output to data through a distance function, $\Delta(D^{*}, D)$, with acceptance determined by a decreasing threshold, ϵ_t, schedule ($\Delta(D^{*}, D) < \epsilon_t$), which is determined automatically. The algorithm proceeds until the fractional change is less than 0.5% ($(\epsilon_t - \epsilon_{t-1})/\epsilon_{t-1} < 0.01$) when we judge the fitting to have converged.

The distance function relates the model output and simulated data D^{*} to the biological data D and here we use the Euclidean distance, given by the expression

$$\Delta(D^{*}, D) = \sum_{d=1}^{d=8} \sum_{k \in T_d} \sqrt{(D_k^d - {D^{*}_k}^d)^2},$$

where T_d is the set of time points that are observed.

In the resampling step, each new particle is sampled from the previous population with a weighted probability and then perturbed with a uniform perturbation kernel. For the special case of the hierarchical model, we perturb only the hyperparameters, γ, with the K values sampled from the corresponding lognormal distributions. The population size (number of particles) was 500 throughout. Model simulation was performed on Graphics Processing Units (GPUs) using the cuda-sim package [6].

5 Model selection using approximations to the AIC and DIC

We used the DIC and AIC to compare models containing one, two and three repair processes [7]. In both cases the lower the metric, the more appropriate the model. Since we cannot write down the likelihood for our stochastic model, we approximate the AIC and DIC using the idea of a surrogate likelihood function [8]. We chose to examine both the AIC and DIC since they assess slightly different measures of fit, depending on whether the K or the γ are the focus.

5.1 AIC

The AIC is given by the expression

$$AIC = -2\log p(D|\bar{\gamma}) + 2p_\gamma \tag{19}$$

where $\bar{\gamma}$ is the mean value of the hyperparameter posterior, and p_γ is the number of hyperparameters. The first term can be written

$$\begin{aligned} -2\log p(D|\bar{\gamma}) &= -2\log \int p(D|K)p(K|\bar{\gamma})dK \\ &= -2\log \int\int p(D|D^{*})p(D^{*}|K)p(K|\bar{\gamma})dK dD^{*}, \end{aligned} \tag{20}$$

where D^{*} are simulated data sets and $p(D|D^{*})$ is the surrogate likelihood function given by

$$p(D|D^{*}) = \frac{1}{\sqrt{2\pi}} e^{-\frac{\Delta(D^{*},D)}{2}}. \tag{21}$$

5.2 DIC

The deviance is given by the expression

$$\text{Dev}(\gamma) = -2\log p(D|\gamma),$$

and we define the quantities

$$\text{Dev}(\bar{\gamma}) = -2\log p(D|\bar{\gamma})$$
$$\overline{\text{Dev}} = E_{\gamma|D}[\text{Dev}(\gamma)],$$

where again $\bar{\gamma}$ is the mean value of the hyperparameter posterior. The Deviance Information Criterion [7] is then given by

$$DIC = 2\overline{\text{Dev}} - \text{Dev}(\bar{\gamma}), \tag{22}$$

where $\text{Dev}(\bar{\gamma})$ is given by Equation 20 and $\overline{\text{Dev}}$ is given by

$$\overline{\text{Dev}} = E_{\gamma|D}\left[-2\log\int\int p(D|D^*)p(D^*|K)p(K|\bar{\gamma})dK\,dD^*\right] \tag{23}$$

References

[1] Juliane Liepe, Chris Barnes, Erika Cule, Kamil Erguler, Paul Kirk, Tina Toni, and Michael P.H. Stumpf. ABC-SysBio–approximate Bayesian computation in Python with GPU support. *Bioinformatics*, 26(14):1797–1799, 2010.

[2] Juliane Liepe, Paul Kirk, Sarah Filippi, Tina Toni, Chris P Barnes, and Michael P H Stumpf. A framework for parameter estimation and model selection from experimental data in systems biology using approximate Bayesian computation. *Nature protocols*, 9(2):439–456, February 2014.

[3] Tina Toni, David Welch, Natalja Strelkowa, Andreas Ipsen, and Michael P H Stumpf. Approximate Bayesian computation scheme for parameter inference and model selection in dynamical systems. *Journal of the Royal Society, Interface / the Royal Society*, 6(31):187–202, February 2009.

[4] Tina Toni and Michael P H Stumpf. Simulation-based model selection for dynamical systems in systems and population biology. *Bioinformatics*, 26(1):104–110, January 2010.

[5] Pedro Mendes, Stefan Hoops, Sven Sahle, Ralph Gauges, Joseph Dada, and Ursula Kummer. *Computational Modeling of Biochemical Networks Using COPASI*, pages 17–59. Humana Press, Totowa, NJ, 2009.

[6] Yanxiang Zhou, Juliane Liepe, Xia Sheng, Michael P. H. Stumpf, and Chris Barnes. GPU accelerated biochemical network simulation. *Bioinformatics*, 27(6):874–876, March 2011.

[7] David J. Spiegelhalter, Nicola G. Best, and Bradley P. Carlin. Bayesian measures of model complexity and fit. *Journal of the Royal Statistical Society, Series B*, 2002.

[8] Francois Olivier and Laval Guillaume. Deviance information criteria for model selection in approximate bayesian computation. *Statistical Applications in Genetics and Molecular Biology*, 10(1):1–25, 2011.

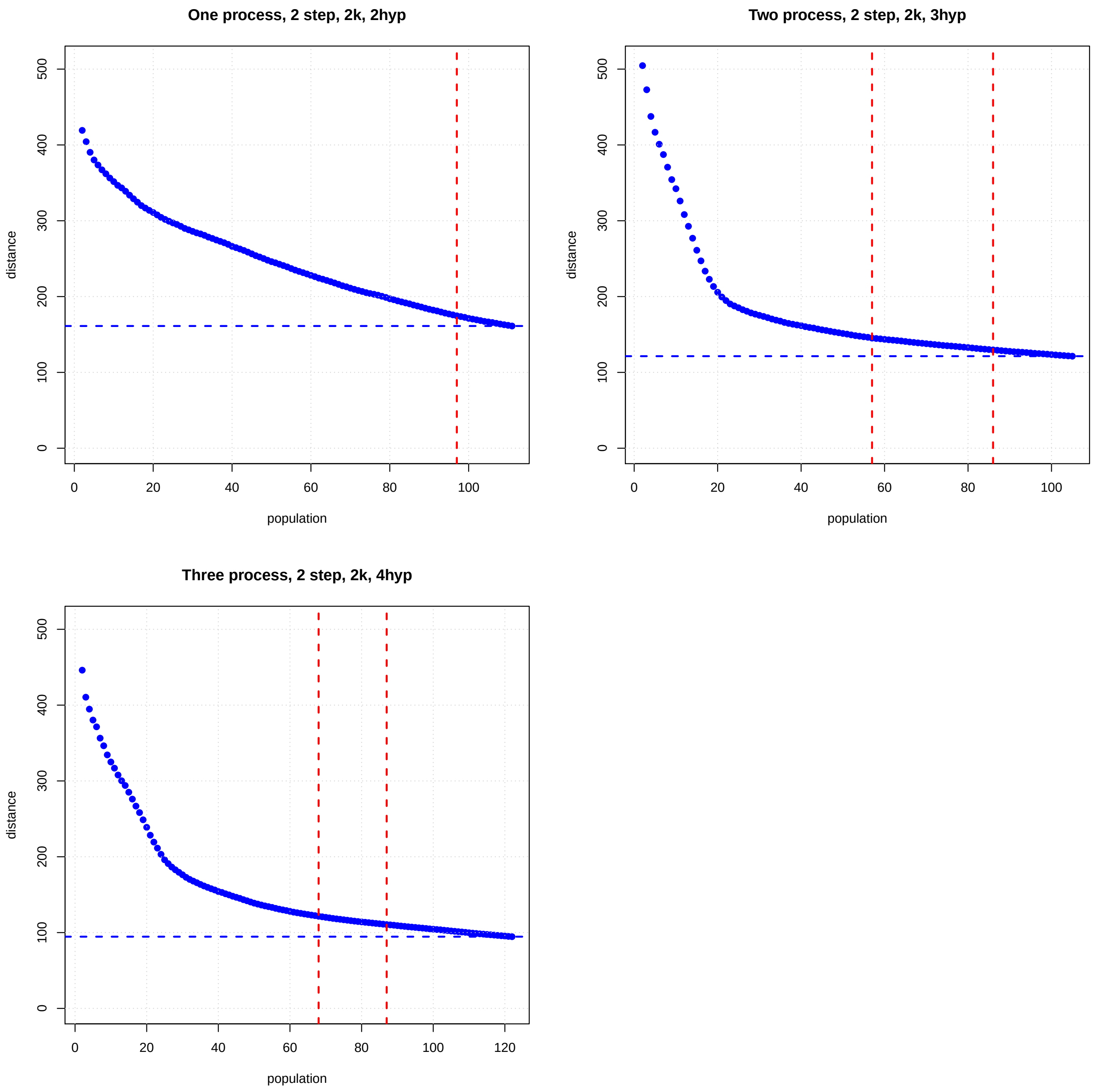

Figure A: Distance against population number for the three models. Blue dashed line is the lower bound of the distance. Red dashed lines represents the point at which the population number percentage change is less than 1% and 0.5%.

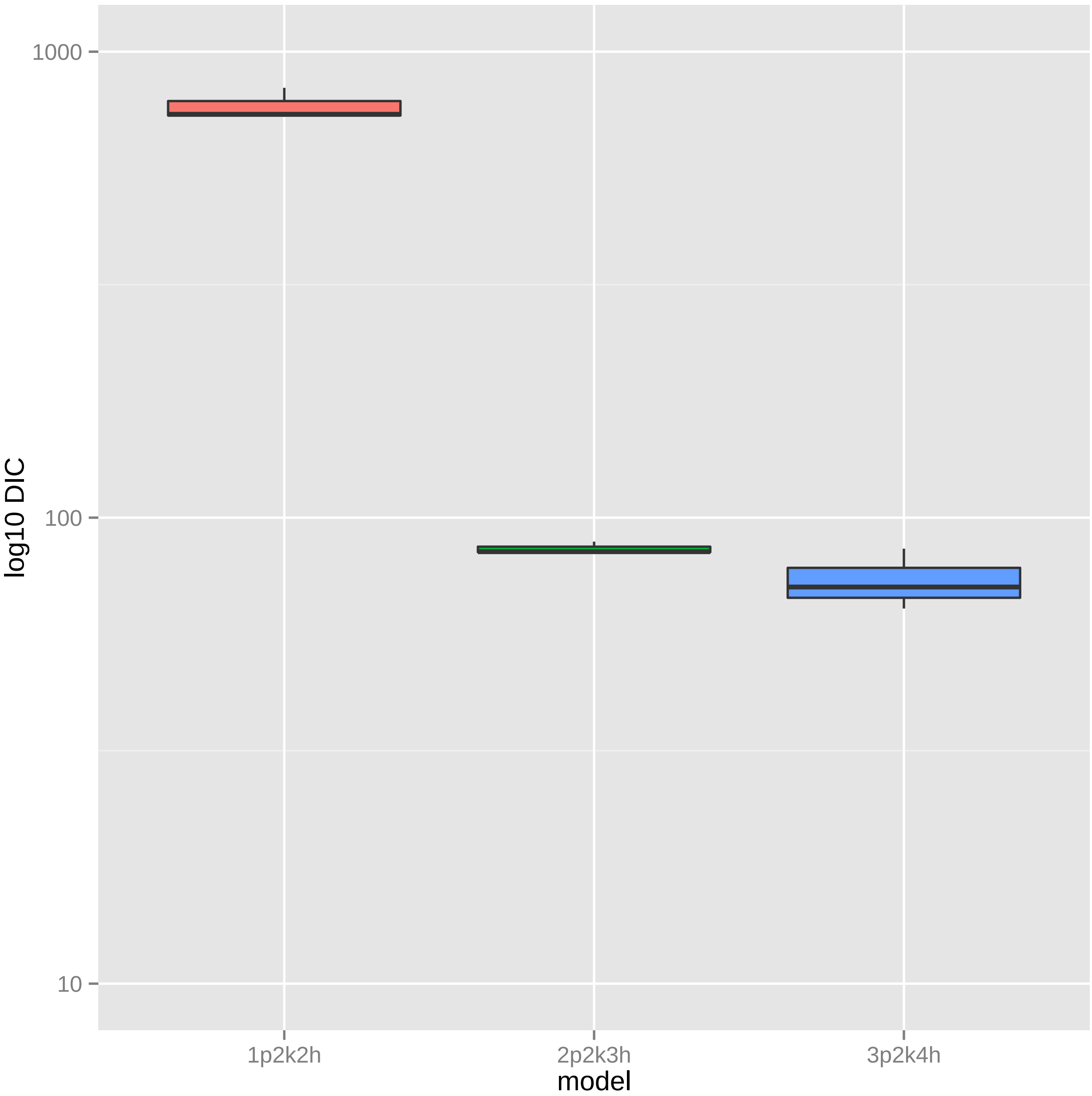

Figure B: Deviance information criterion for the models M_1, M_2 and M_3. The three process model gives the best score.

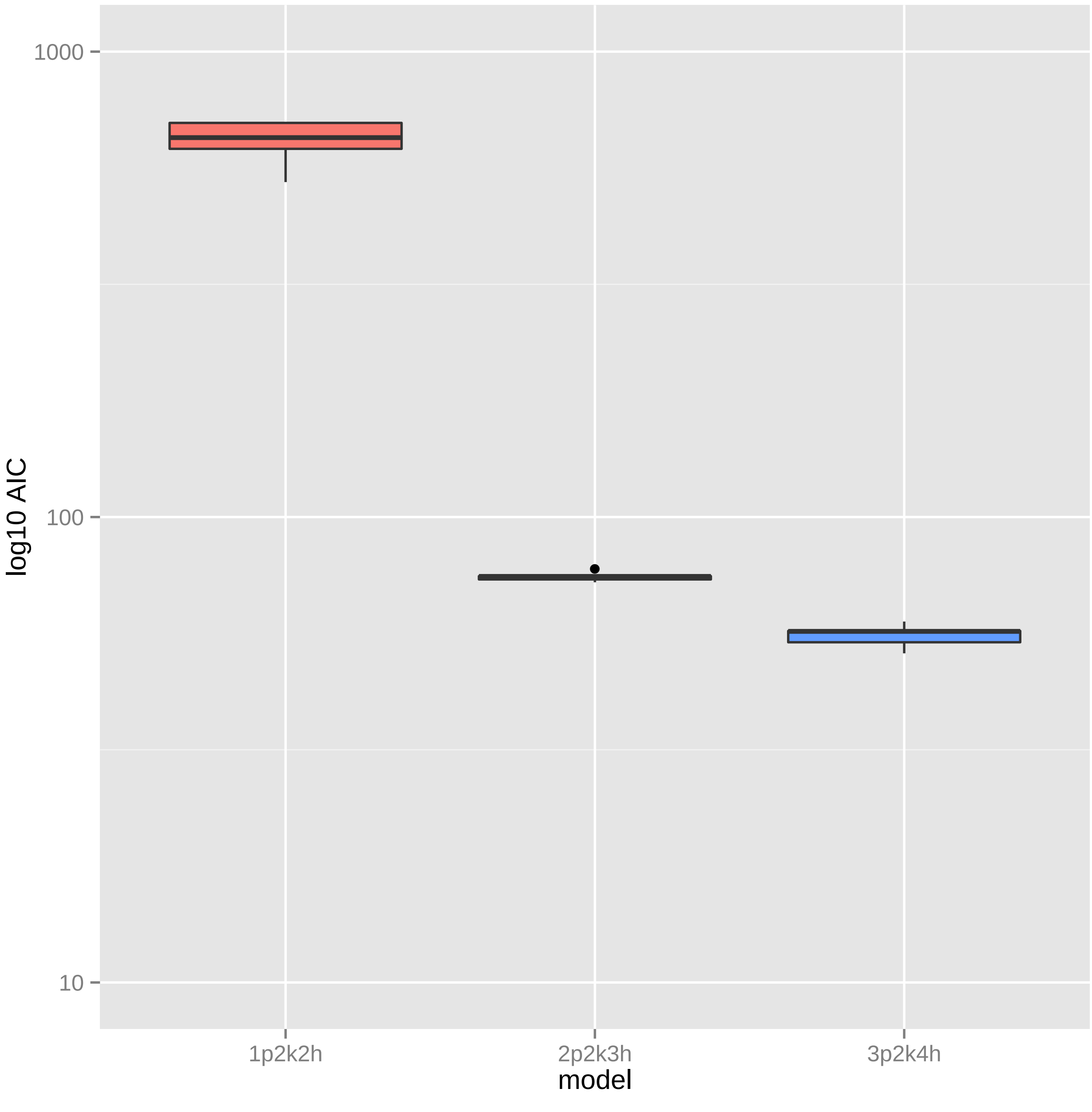

Figure C: Akaike information criterion for the models M_1, M_2 and M_3. The three process model gives the best score.

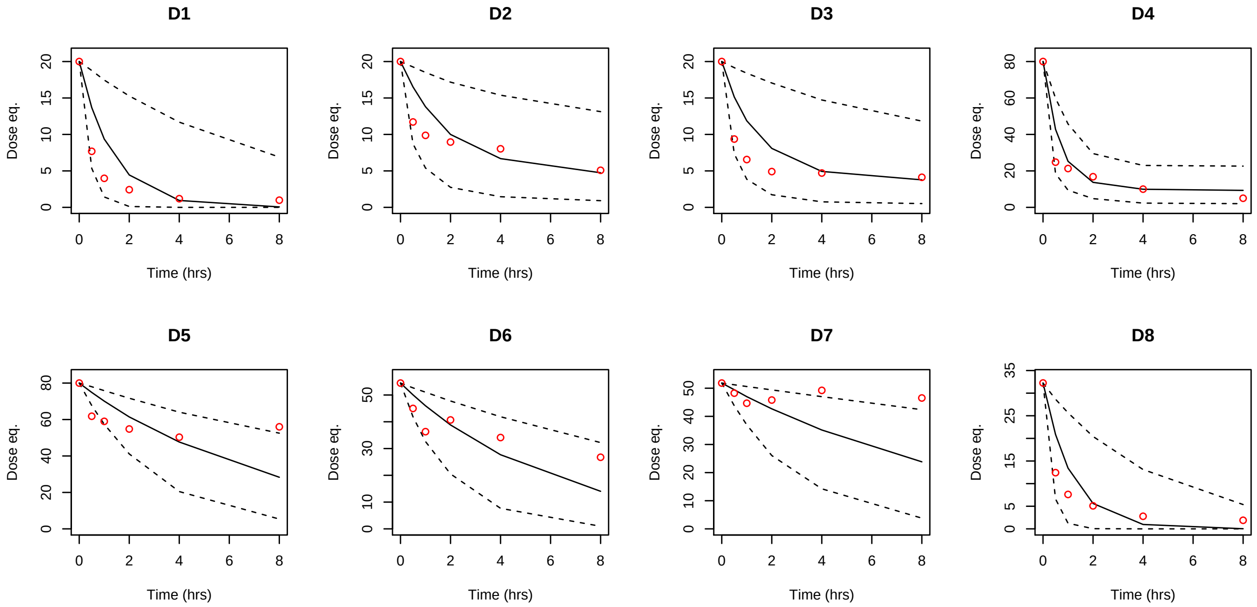

Figure D: Trajectories from the posterior of the one process model M_1.

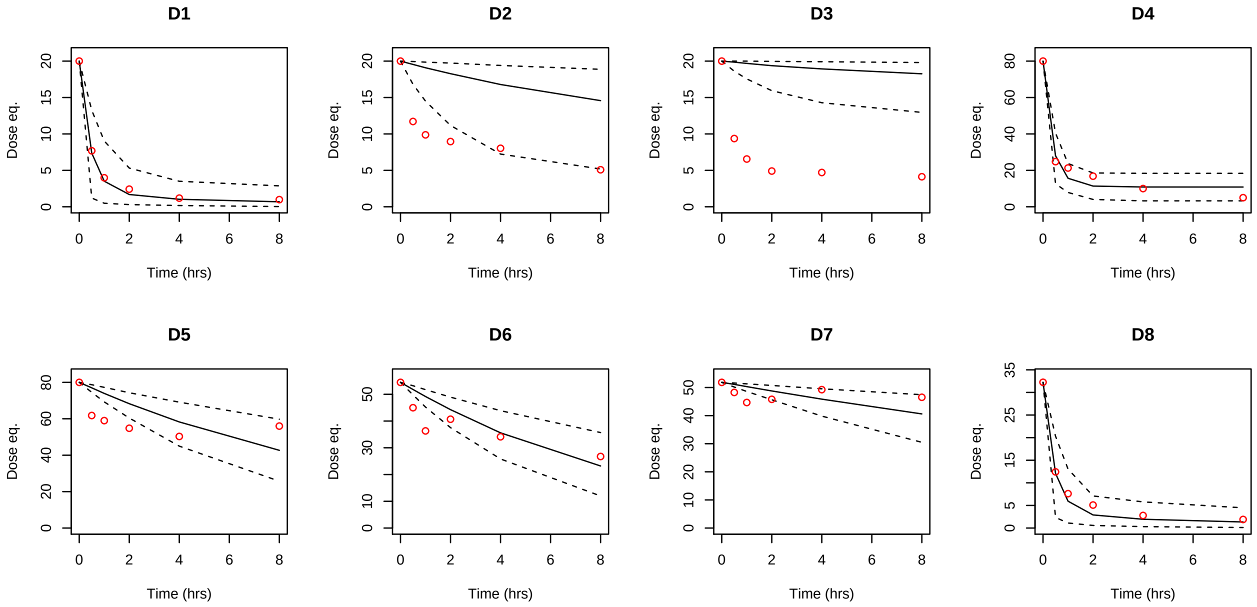

Figure E: Trajectories from the posterior of the two process model M_2.

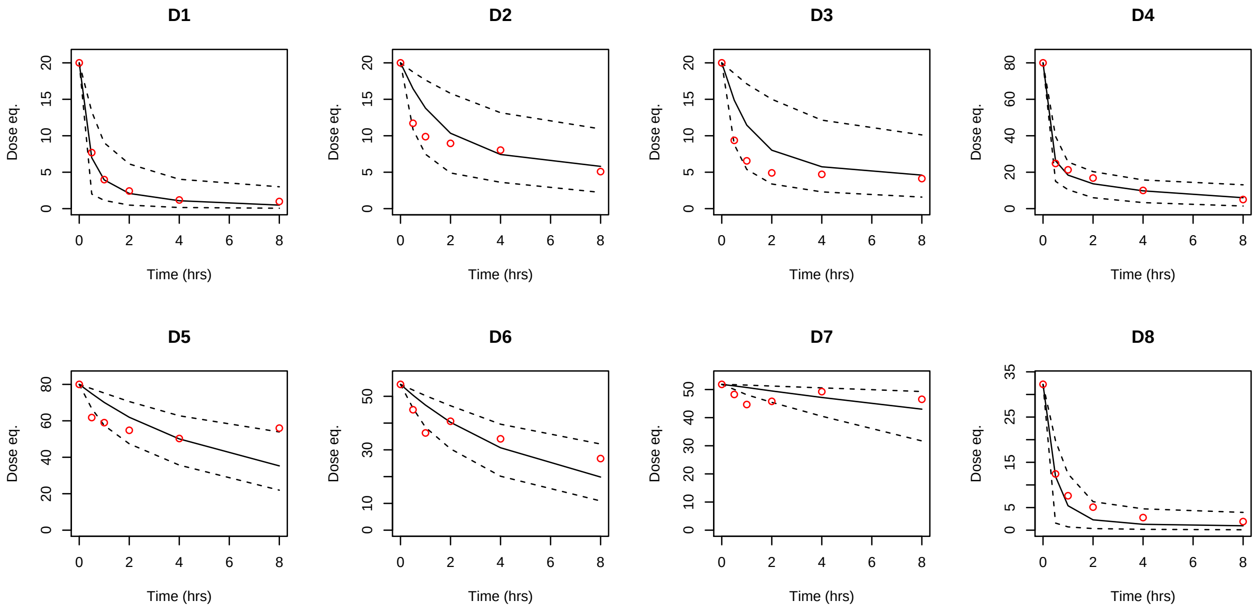

Figure F: Trajectories from the posterior of the three process model M_3.

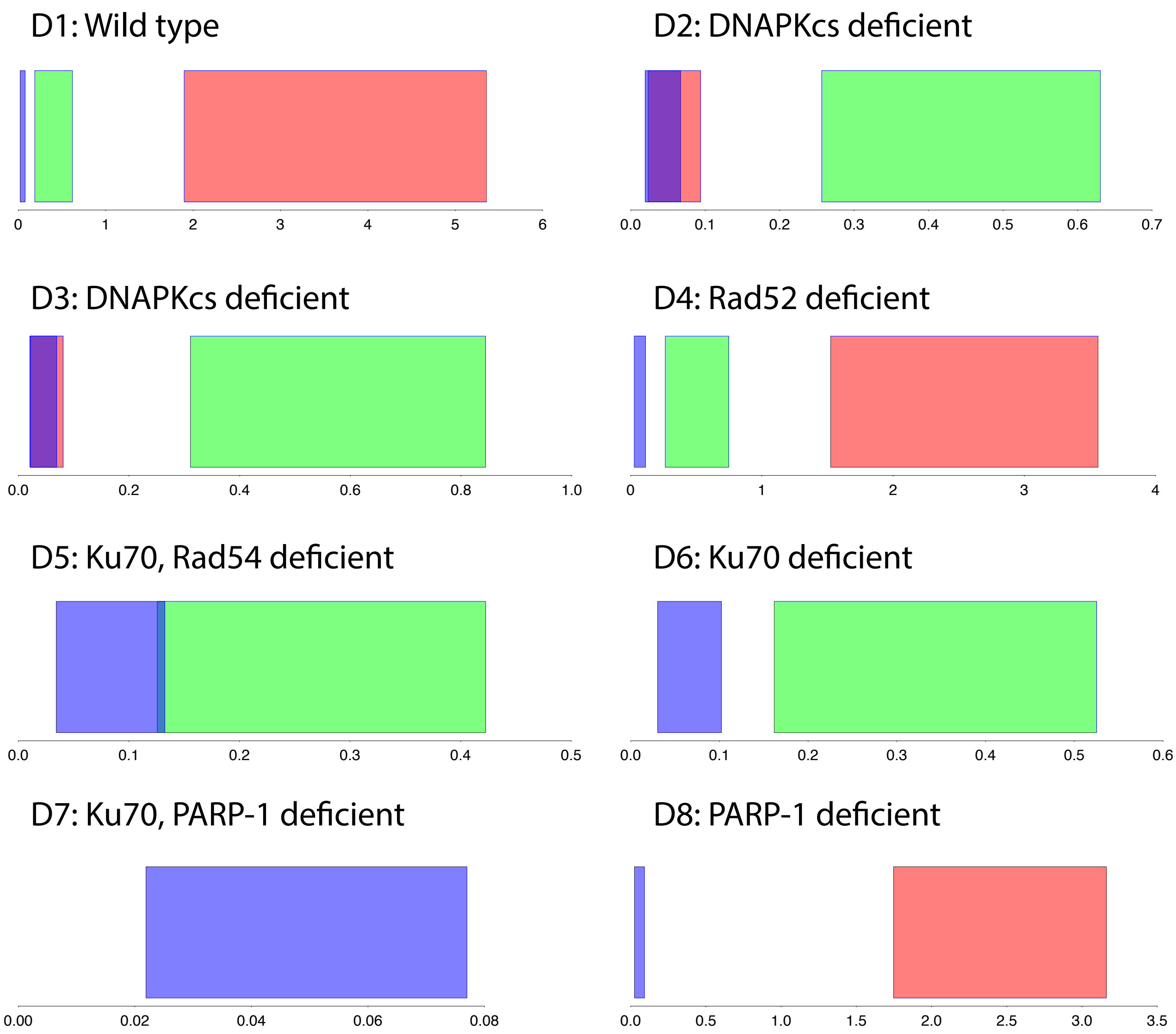

Figure G: The interquartile range for all the parameters K_i in each dataset. Red (fast repair), blue (slow repair) and green (intermediate repair).

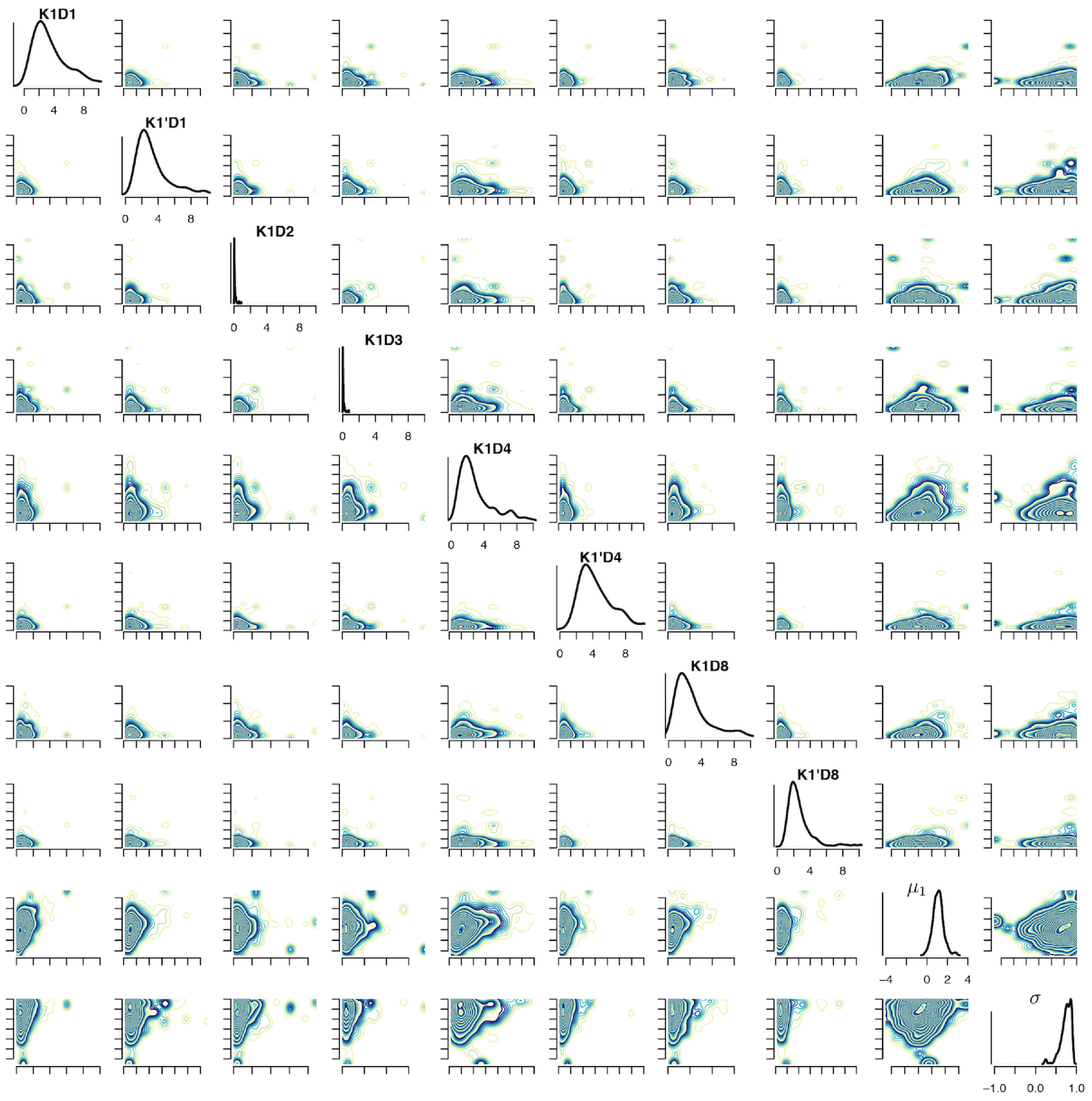

Figure H: Univariate and marginal distributions of the posterior for NHEJ.

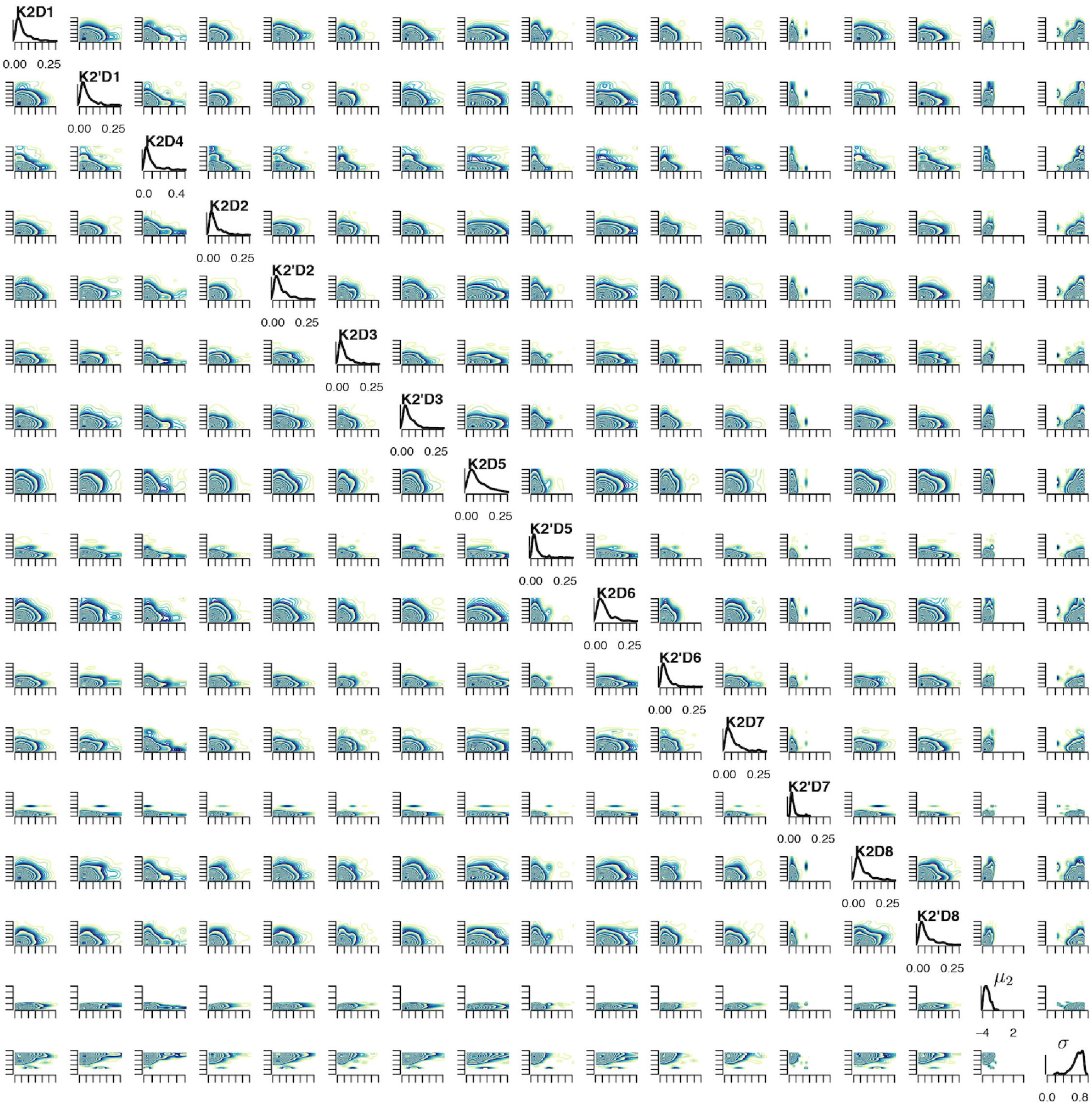

Figure I: Univariate and marginal distributions of the posterior for SSA.

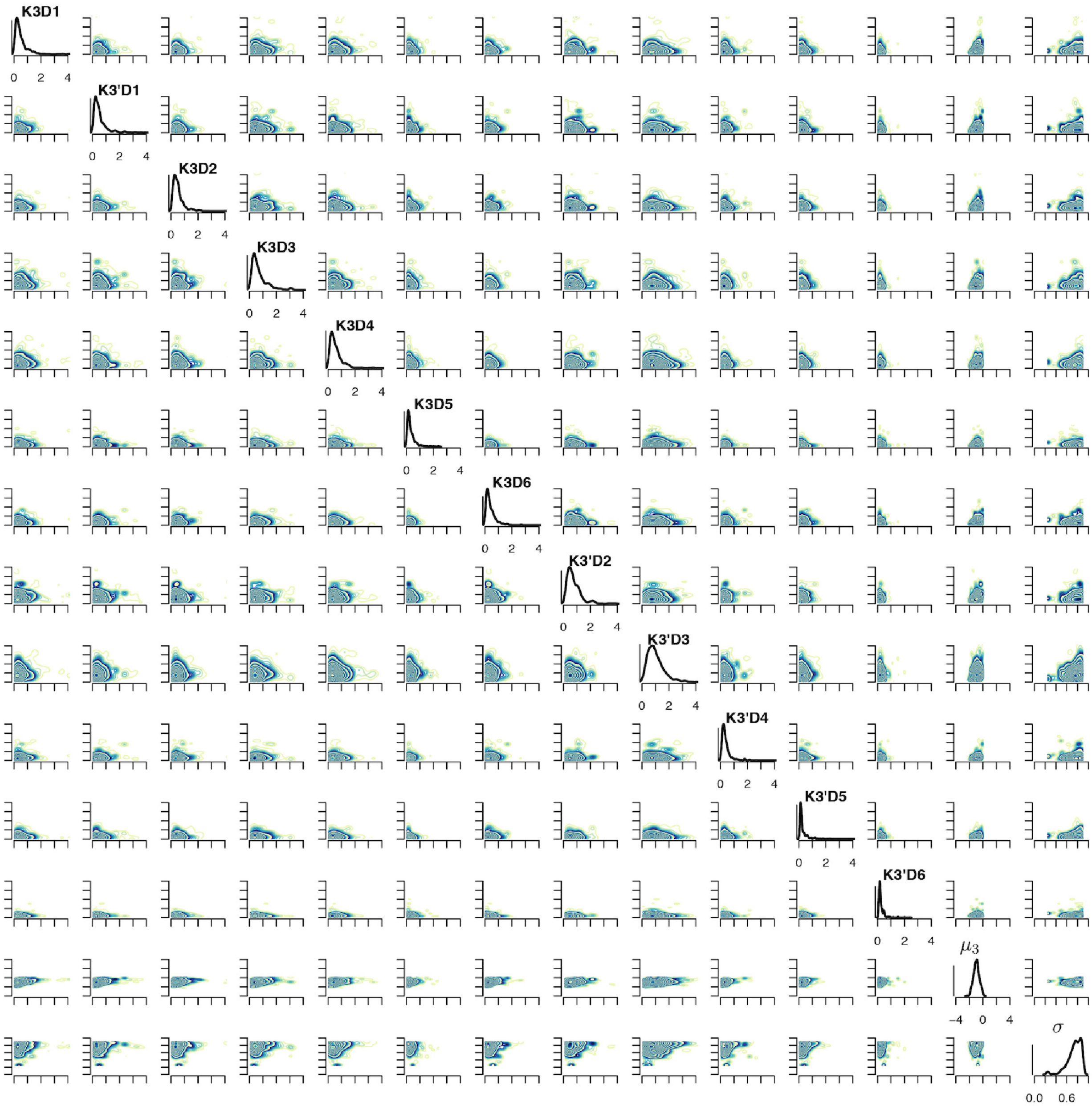

Figure J: Univariate and marginal distributions of the posterior for A-EJ.

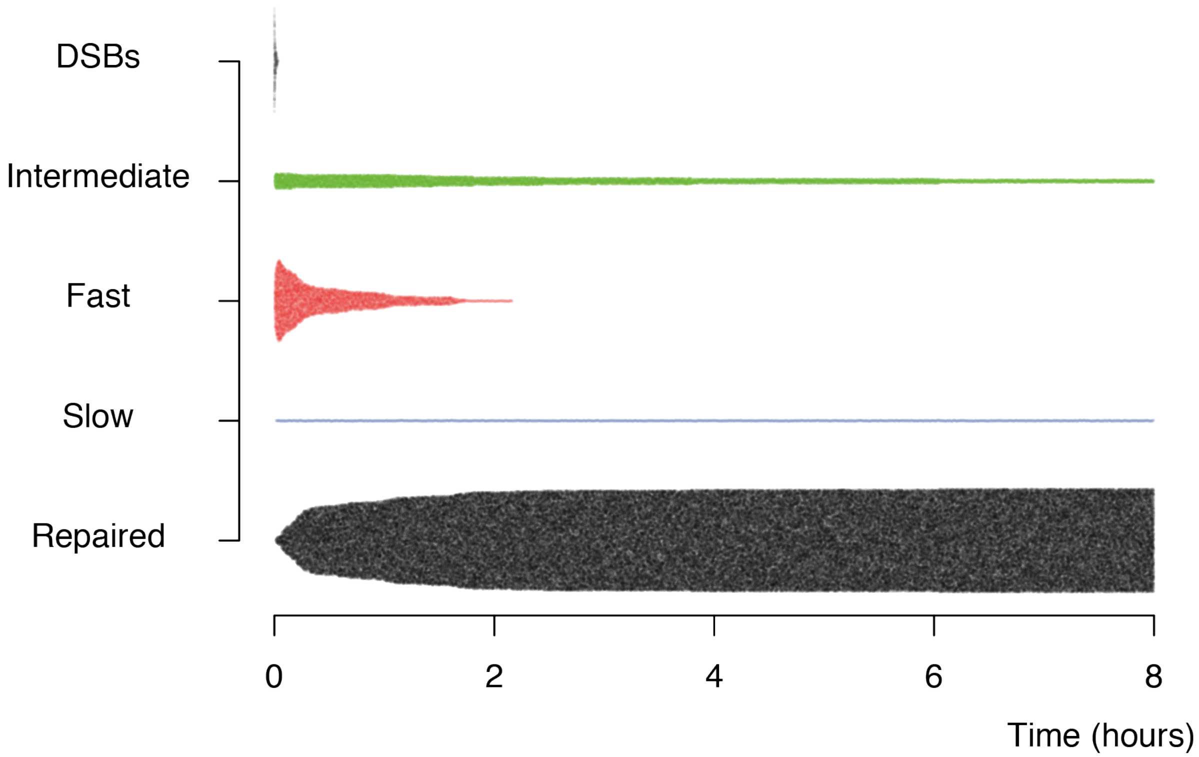

Figure K: Time series showing a typical distribution of individual DSBs from one simulation.

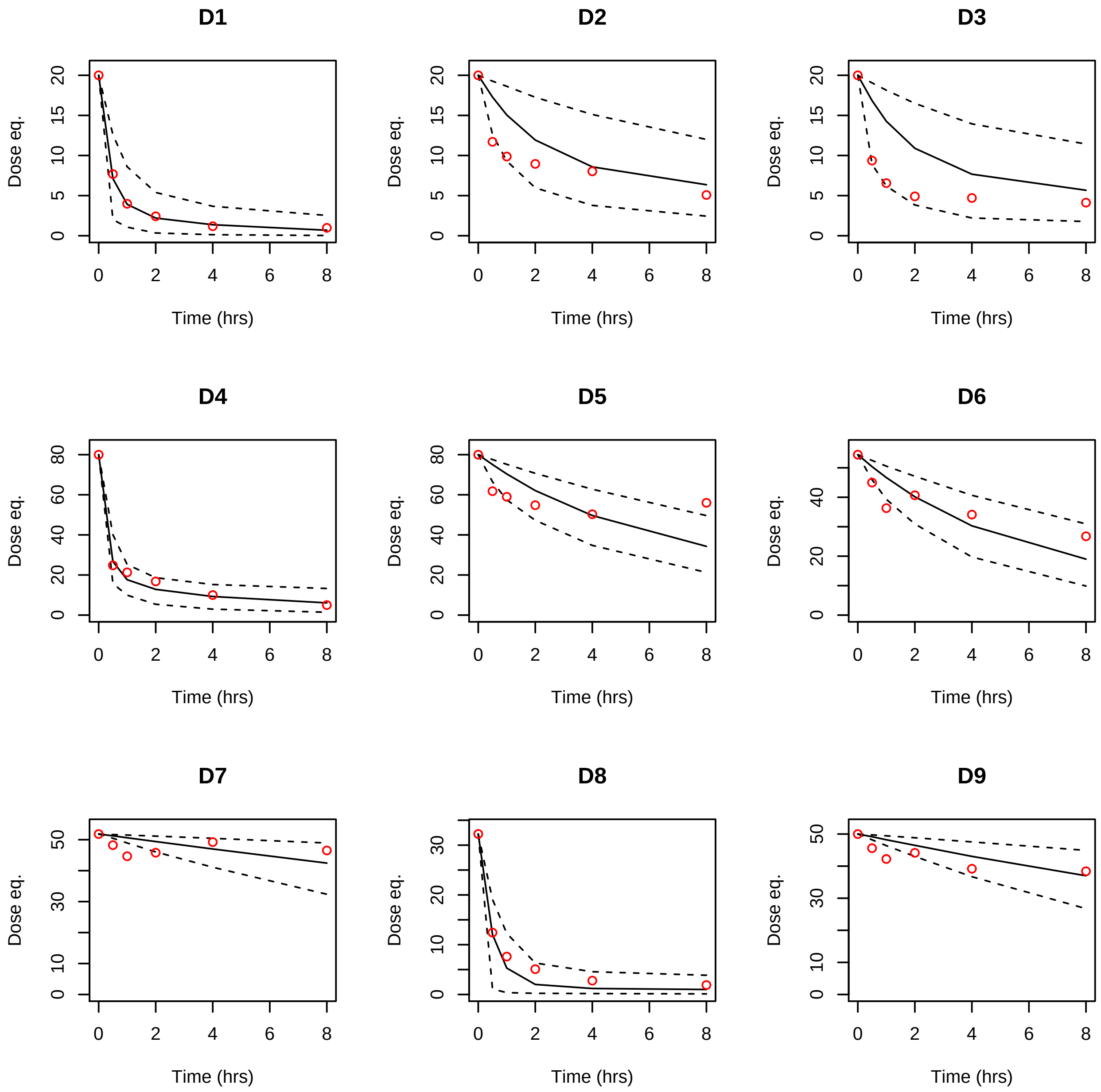

Figure L: Time series showing the model fit for 8 datasets, including an additional dataset for xrs-6 cells with inhibition of PARP-1 with DPQ.

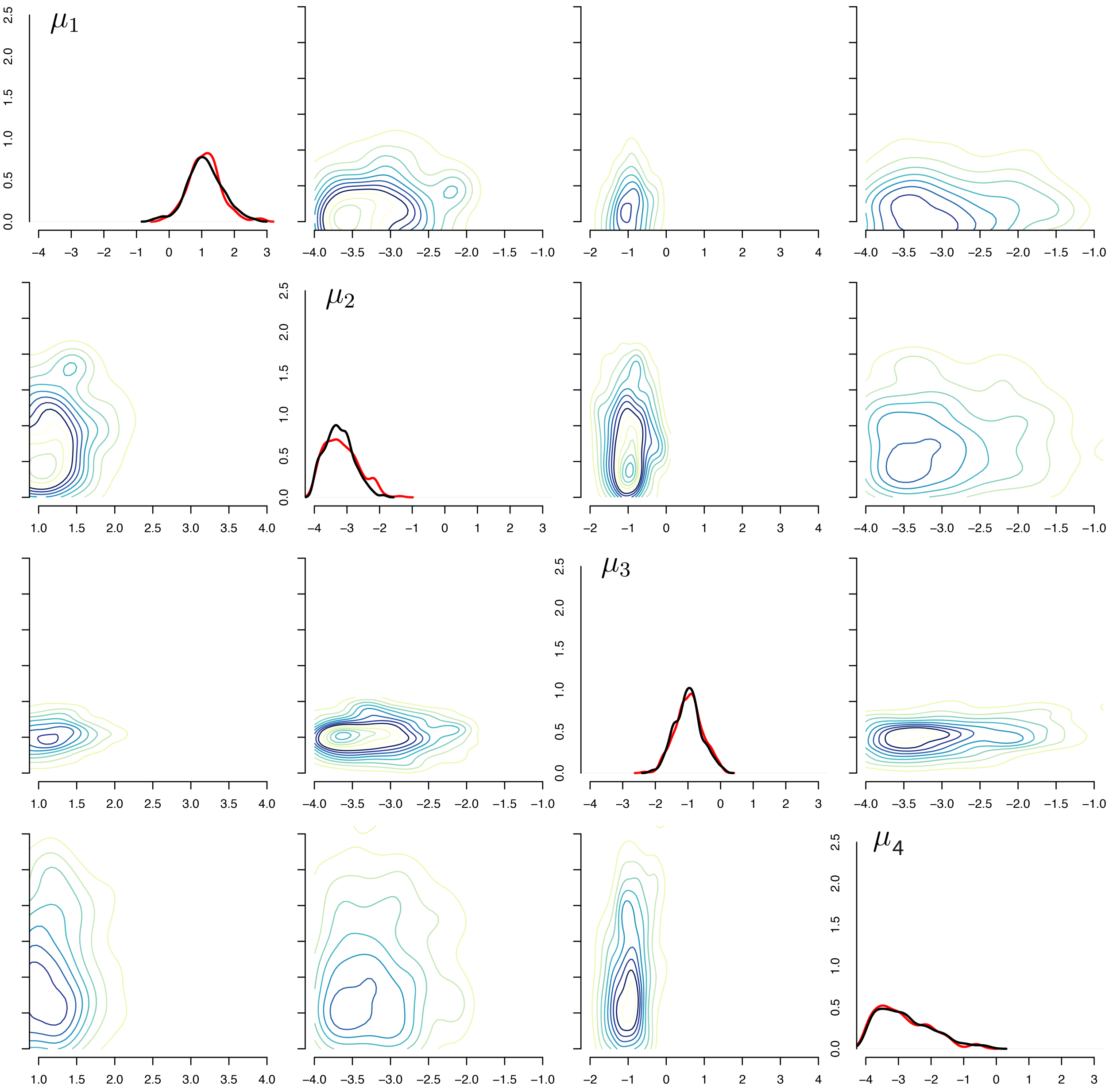

Figure M: Posterior distributions of the hyper parameters μ_i for the eight dataset model (black lines, diagonal plots). Posterior distributions of the hyper parameters μ_i and the marginal distributions for the nine dataset model (red lines).

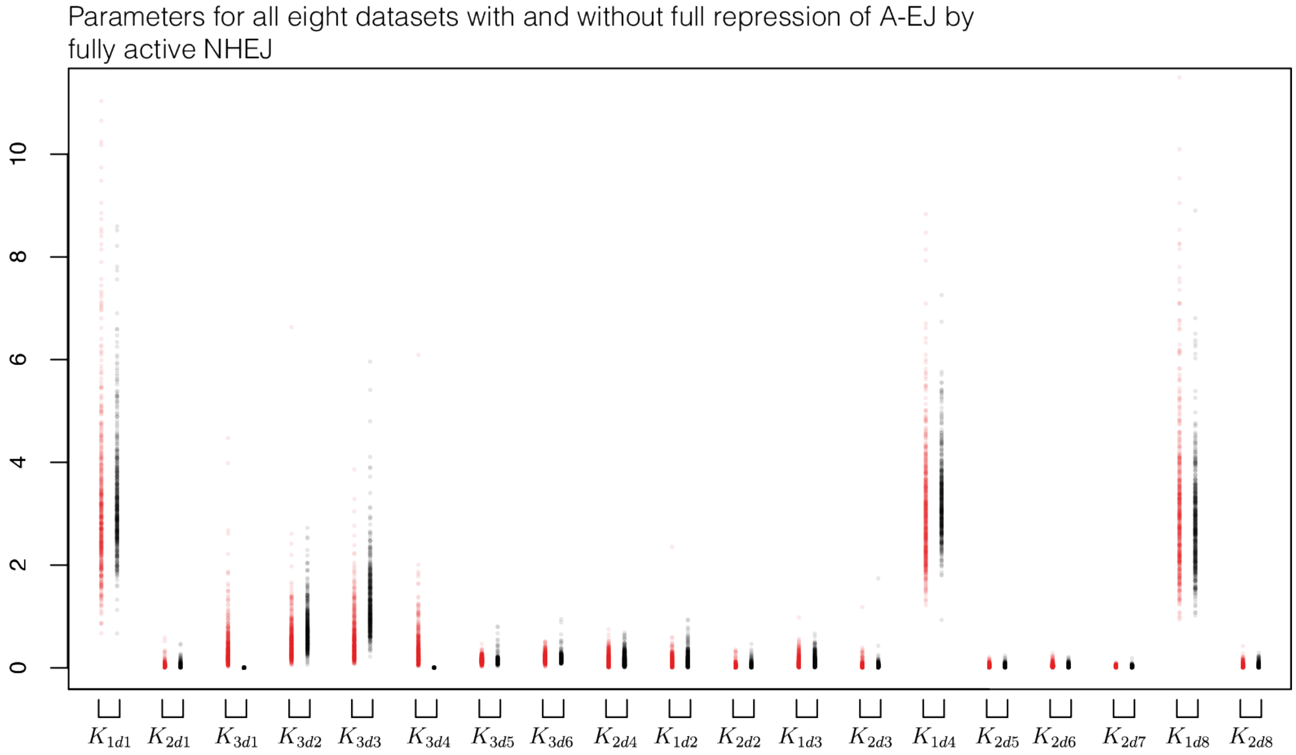

Figure N: Distribution for each parameter K_i for the eight dataset model (red points), compared with parameter distribution for full repression of A-EJ by active NHEJ (black points).
